# Plant polyphenols effect on gut microbiota: Recent advancements in clinical trials

**DOI:** 10.17179/excli2021-3900

**Published:** 2021-06-09

**Authors:** Ayshwarya Lakshmi Chelakkot, Cijo George Vazhappilly

**Affiliations:** 1 Independent scholar; 2 Department of Biotechnology, American University of Ras Al Khaimah, Ras Al Khaimah, United Arab Emirates

## ⁯⁯

***Dear Editor,***

The diverse microbial community present in the human intestine plays a vital role in translating the food to nutrients and metabolites essential for maintaining host physiology, including digestion, lipid and glucose metabolism, immune homeostasis, and proper development of the brain and cognitive functions (Klinder et al., 2016[13]; Park et al., 2020[18]). Altering the gut microbiota through dietary interventions for the prevention or treatment of disorders might lead to developing a novel concept called 'personalized nutrition' and help better understand the effects of dietary bioactive compounds on the host microbiome. 

Recent research and clinical trials have identified the beneficial effects of a plant-based diet to increase bacterial diversity and ameliorate various disorders, including intestinal disorders, obesity-related endotoxemia, and cardiovascular disorders (Vazhappilly et al., 2019[22], 2021[21]; Guglielmetti et al., 2020[9]). Fruits and berries are rich in polyphenols and modulate gut microbiota by increasing the global fecal bacteria (Klinder et al., 2016[13]; Moreno-Indias et al., 2016[16]; Teixeira et al., 2017[20]; Ntemiri et al., 2020[17]; Rahman et al., 2021[19]). For instance, in obese and overweight people, a change in the gut microbiota, with a consequent decrease in endotoxemia, through probable modulation of the *Faecalibacterium*, *Odoribacter*, and *Parvimonas*, was noted on consuming pomegranate extract (González-Sarrías et al., 2018[7]). Consumption of two SunGold kiwi fruits per day increased plasma vitamin C and fasting glucose significantly, decreased HbA1c levels, and improved cardiovascular and metabolic markers (Wilson et al., 2018[25]). Modulation of gut microbiota using red wine also showed protective effects on obesity-related metabolic disorders (Moreno-Indias et al., 2016[16]). The polyphenols in cocoa powder and green tea, especially flavanols epicatechin and catechin, are metabolized by the microbiota with increased bioavailability and similar protective outcomes (Janssens et al., 2016[12]; Gómez-Juaristi et al., 2019[6]; Ángel García-Merino et al., 2020[1]; Vilela et al., 2020[24]). A deeper understanding of the correlation between dietary metabolites and gut microbiota is, therefore, essential to attain beneficial effects of modulating host-microbiome under disease conditions. The below table(Tab. 1) (References in Table 1: Ángel García-Merino et al., 2020[1]; Basak et al., 2020[2]; Chashmniam et al., 2019[3]; Conterno et al., 2019[4]; de Oliveira Silva et al., 2020[5]; Guevara-Cruz et al., 2020[8]; Hidalgo-Liberona et al., 2020[10]; Istas et al., 2019[11]; Lima et al., 2019[14]; Medina-Vera et al., 2019[15]; Ntemiri et al., 2020[17]; Park et al., 2020[18]; Vetrani et al., 2020[23]; Vilela et al., 2020[24]) summarizes the recent clinical findings on the relation between various dietary polyphenols and gut microbiota.

## Acknowledgement

The authors thank the American University of Ras Al Khaimah for the support and facilities provided.

## Conflict of interest

The authors declare no conflict of interest.

## Figures and Tables

**Table 1:**
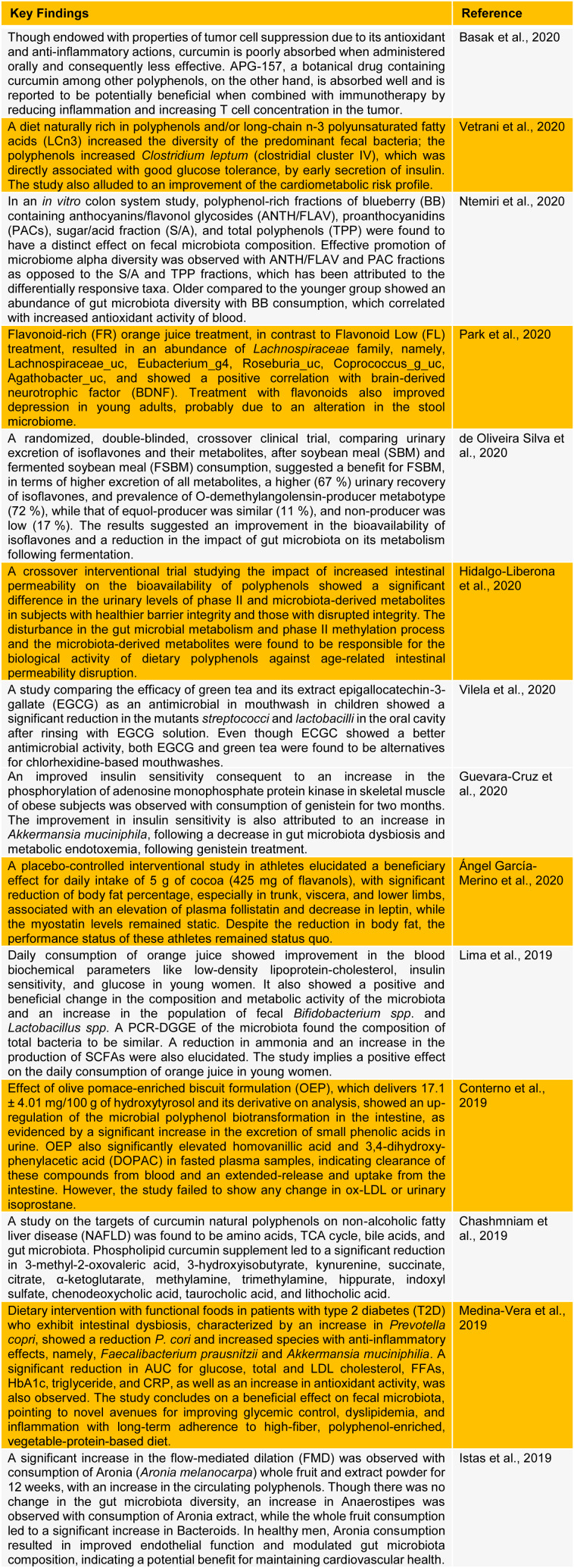
Table1: Correlation between plant polyphenols and gut microbiota
